# Digital citizens’ feelings in national #Covid 19 campaigns in Spain

**DOI:** 10.1016/j.heliyon.2021.e08112

**Published:** 2021-10-06

**Authors:** Sonia Santoveña-Casal, Javier Gil-Quintana, Laura Ramos

**Affiliations:** Department of Didactics, School Organization and Special Education, National University of Distance Education, 28020 Madrid, Spain

**Keywords:** Digital citizenship, Twitter, e-participation, Covid-19, Discourse analysis

## Abstract

**Background:**

In 2020 Spain launched an official campaign, #EsteVirusLoParamosUnidos, aimed at uniting the entire country through citizen cooperation, in order to combat Covid-19. The objective of this research has been to analyse how this Twitter campaign revealed the feelings expressed by Spanish citizens.

**Methods:**

The research is based on a composite design that triangulates, from a theoretical model, a quantitative analysis and a qualitative analysis.

**Results:**

Of the 7,357 tweets in the sample, 72.32% were found to be retweets. Four content families were extracted which relate to politics, education, messages to society and the defence of occupational groups. The feelings expressed ranged from those of unity, admiration and support to those of discontent and criticism of issues regarding the health situation.

**Conclusions:**

The development of networked socio-political and technical measures, which enabled citizen participation, facilitated the development of new patterns of interaction between national or regional governments and digital citizens. This increased citizens’ possibilities of influencing the public agenda and, therefore, strengthening citizen engagement regarding specific situations.

## 1 Introduction

In 2020 the Covid-19 pandemic affected society worldwide and had a direct impact on governments and citizens alike. Social contexts of all kinds underwent sudden changes, lockdown restrictions met with resistance. Rates of infection, death tolls and news of a possible economic crisis sowed uncertainty. In response, governments not only rolled out health and safety measures, but also launched citizen awareness campaigns, choosing the Internet and social networks as their medium of choice.

In our increasingly high-tech digital society governments everywhere have been implementing socio-technical measures [1] to deal with the various socio-political and economic challenges facing them [2]. This is facilitating the creation of new connections and patterns of interaction between governments, citizens and technology [3]. Never before has it been so important to rely on the participation of citizens to solve a public health problem. In fact, an increase in awareness that citizen participation can contribute to, and support, public health policies has been observed [4]. The earliest examples of this can be found in those government initiatives, aimed at strengthening digital participation through deliberation and decision-making processes, which enable citizens to decide how public spending is allocated (electronic participatory budgeting). Governments worldwide, and official administrations in cities like New York, Madrid and Paris, run electronic participatory budgeting initiatives [5].

The concept of digital citizenship [6, 7] has many meanings. It can be viewed from a technology-based perspective or from one which includes socio-political elements [8, 9]. It evolves from a focus on digital components to an ethical and responsible use of technology [10] as well as an emphasis on critical, civic, and daily citizen participation [11]. In this paper we deal with the concept of democratic citizenship which includes responsible citizenship [12]. This involves actions relating to social and political participation and is oriented toward responding to those social and economic problems which are often outside the canons of the traditional concept of citizenship [13].

Social networks not only enable interaction and communication between different spheres [14, 15, 16], but also directly influence government decisions and even challenge laws and legislative changes [17]. In fact, it has been observed that the Internet may influence citizens’ civic commitment [18, 19] in the development of participatory processes and by affecting the development of interaction and communication between the political agenda and citizens.

Within this context citizens are able to organize, and challenge, the official line or dominant discourse and governments may counter these challenges by posting arguments of their own. Governments, social movements and public institutions the world over have been seen to use networks to communicate with society [20]. The data show, for example, that international public administrations with responsibilities for health have Twitter profiles [21].

## 2 Social networks and COVID-19

Social networks make it easier to express and mobilize emotions, as well as share information with the community [22]. Emotions become acts for sharing, and social media offers public forums where individuals can express sadness and feelings related to painful situations. This gives visibility and publicity to pain and loss [23]. As a result of recognising the influence of Twitter on our lives, several apps have been developed in order to detect messages from people who are depressed, or those with suicidal tendencies [24]. Emotions are tracked through different parameters, including the location or the date on which the tweet was sent.

Twitter has been regarded as an extremely important social network throughout the Covid-19 health emergency [25]. It is used, more than any other network in Spain, to discuss socio-political affairs [26]. Furthermore, the government has been observed to use Twitter to spread information related to administrative issues and press conferences, although without facilitating any true interaction between government and citizens [27, 28].

During the last year, multiple investigations have been published focussing on how the use of the social network Twitter has been correlated to different aspects of Covid-19. This includes the impact of lockdown on citizens [29], the detection of warning signs about the initial outbreaks of the Covid-19 [30], the effect of risk perception on social distancing [31], information on aerosols [32], the relationship between topics discussed on the platform and the feelings they generate [33], misinformation [34], Twitter as a tool for communication between doctors [35], communication between people with different diseases during the pandemic [36] and alcohol consumption during the pandemic [37].

## 3 Twitter, emotions and COVID-19

The analysis of feelings is an area which studies the opinions, ideas and emotions publicly expressed through social networks like Twitter. Feelings are considered imperative in order to ‘judge human behaviour’ [38]. Sentiment analysis, through the data provided by Twitter, has been explored from different perspectives and aimed at different objectives. It can be seen that these data are polarized (positive-negative) and subjective [39]. A number of research projects have focused on the analysis of emotions, not only in order to understand the development of social reality, but also to know how to solve a number of social problems [40]. The rapid growth of data in social networks has necessitated the development of different Big Data Analytics (BDA) systems. Within the framework of the text mining, and when analysing social networks, the following systems have been used: Support Vector Machine, Qualitative content analysis, Principal Component Analysis, Clustering, Descriptive Analysis, Imputation method, Clustering, Emotional Co-Creation Score, Clustering, Emotional Text Mining, Sentiment Analysis, Emotional analysis, Network analysis [41].

The most frequently-used techniques for the analysis of emotions on Twitter have been Machine Learning and Lexicon-based techniques [42, 43]. Machine Learning models [44] is a fundamental method which needs to be taken into consideration when undertaking an analysis of emotions expressed on Twitter [45]. However, the Lexicon-based method is based on the use of dictionaries and terms based on emotion (positive, negative or neutral) [46].

The Valence Aware Dictionary for Sentiment Reasoning (VADER), Python [47], R's Syuzhet library [48, 49, 50] are powerful tools in this methodology. Here, the variables of language must be considered. For example, there are studies focused on messages published in Arabic [51], Portuguese and English [52] and in Greek [53].

The study of emotions on Twitter has generated a large volume of research [54, 55]. During the pandemic, the relationship between the evolution of Covid-19 and emotions has been analysed [56]. Research from 2020, based on more than 20 million tweets, evidenced four predominant emotions: fear (owing to the scarcity of medical supplies), anger (due to the Covid-19 lockdown), sadness, (resulting from personal losses) and, finally, joy and gratitude for good health [57]. Other research projects, involving 24,000 tweets, indicate that, despite the presence of negative feelings (fear, anger, sadness…), the predominant feeling was positive.

At the beginning of the health crisis, some studies revealed the preponderance of positive or neutral feelings [58]. However, as the crisis worsened, the emotions expressed on Twitter were predominantly negative [59, 60]. These authors assert that the negative evolution of the pandemic could have been the cause of an increase in negative feelings. It seems that fear has been the most common emotion expressed during the pandemic in addition to sadness about the disease and resulting deaths [53]. A study, carried out by Xue [45], based on an analysis of 1.9 million tweets related to the Covid-19 crisis (from January to March 2020), concludes that the unknown nature of the virus is the aspect that frightened the population the most. For the Chinese population, the greatest number of concerns, which generated a negative sentiment, were related to the origin of the disease, the spread of the virus and public health [61]. Other authors focused on a sentiment analysis undertaken in Singapore. This study found that emotions were mainly positive. However, differences were observed depending on the theme and the evolution of pandemic [47]. An increase in feelings of stress experienced by students was also detected due to the closure of schools [62].

In any case, we consider it of particular interest to explore the opinions, behaviours and emotions of the population before the start of the Covid-19 outbreak [63]. This is because it can lead to the behaviours which are more or less adjusted to the norms imposed in the different countries [64].

In this article we express our belief that research, based on the dissemination of information on Covid-19, via Twitter, can be a helpful tool for public health institutions and governments with regard to communicating protocols for health emergencies and risk situations [65]. Hence the importance of this investigation which aims to shed light on the involvement, and the role, of Spanish citizens during the pandemic within the context of a Covid-19 information campaign promoted, on Twitter, by the Spanish Ministry of Health. This analysis shows an extended engagement by Spanish society in the fight against Covid-19. It is evidenced by public engagement with social networks and in terms of collaboration with the Government. Analysis of Twitter debates reveals aspects related to citizens’ ways of communicating with Government, links developed within these contexts and feelings expressed by Twitter users. Therefore, it is crucial to analyse these new Twitter-based relationships, which exist between institutions and citizens, as well as explore their effectiveness. For this reason, we have analysed the public health campaigns launched by the Spanish government during the pandemic, regarding them as a subject of particular interest. From March to December 2020, the Spanish government used the Internet, especially Twitter, to launch five campaigns related to Covid-19.

The main objective of the campaign was to raise citizens’ awareness of the effects of the virus and to create a feeling of unity between Spanish citizens in the face of this grave situation. The five campaigns were:

1) #EsteVirusLoParamosUnidos (United We'll Stop This Virus),
2) #SalimosMásFuertes (We're Coming Out Of This Stronger),
3) #EstoNoEsUnJuego (This Is No Game),
4) #GripeYoMeVacuno (Flu I'm Getting My Shots) and
5) #ElMejorRegaloEsCuidarnos (Taking Care Of Ourselves Is The Best Gift).

In this article we analyse the #EsteVirusLoParamosUnidos campaign. Its objective has been ‘to unite the efforts of the entire country in the fight against Covid-19 and offer incentives for respecting social distancing measures’ [65]. The overall aim of the research has been to analyse citizens' feelings in general, and how citizens feel in relation to the official Spanish Covid-19 campaign #EsteVirusLoParamosUnidos. The specific objectives are:

1. To study the communication methods used during the discussion generated by the campaign on Twitter.
2. To identify the main themes aired during the public deliberation.
3. To ascertain the prevailing emotions associated with each theme.
4. To examine the connection between citizens' discourse and the social reality citizens were experiencing at the time.

To further investigate the main issue the following questions were asked:

1. What is/was the nature of the general feeling of Spanish citizens regarding the campaign #EsteVirusLoParamosUnidos?
2. Can it be asserted that a debate took place between citizens as a result of this campaign?
3. What topics were aired on Twitter? What feelings were generated by these topics?
4. Is there any connection between citizens' discourse and the social reality experienced at the same time? What's this connection?

## 4 Materials and methods

### 4.1 Population and sample

The #EsteVirusLoParamosUnidos campaign was selected for analysis for two fundamental reasons: first, because it was the Spanish Ministry of Health's first campaign related to Covid-19. Second, because its objective was primarily collaborative, since it sought to unite citizens in the fight against Covid-19. At the same time, it was aimed at strengthening social distancing measures, so as to reinforce citizen engagement when confronting the problem. Furthermore, ‘EsteVirusLoParamosUnidos’ became the Spanish Government's slogan for motivating the population to embrace good practices, crossing the limits of social networks. At the time of data collection, the #EsteVirusLoParamosUnidos hashtag had a higher engagement rate on Twitter than other campaigns. Data from May 2021 shows that the hashtag #EsteVirusLoParamosUnidos attracted 37,636 tweets, in contrast to the hashtag #EstoNoEsUnJuego which attracted just 24,440 tweets. Narrowing the investigation to the hashtag in question allowed for a more in-depth analysis of the content and the different fronts open on Twitter to citizens at a very sensitive and complex period of time for society.

Within this framework this research analyses usage of the Twitter social network in connection with the #EsteVirusLoParamosUnidos hashtag. Tweets were selected from the period spanning 12^th^ September, 2020, to 16^th^ October, 2020. This period was in line with the end of the summer season, the return of workers to their jobs, the reopening of schools and the tightening of restrictive measures in the face of the new surge in Covid-19 cases. It was characterised by high tension within diverse groups, thus generating very relevant interactions for the purpose of our research.

The sample contains a total of 7,357 tweets, after 1,310 tweets were discarded because they failed to offer information related to the topic of study. The original sample therefore contained 8,667 tweets. Among the 7,357 tweets selected, there was a total of 3,675 users who participated in the dissemination of the #EsteVirusLoParamosUnidos hashtag through direct tweets or retweets (RTs).

### 4.2 Design and instruments

The research is based on a composite triangulating design involving a theoretical model, a quantitative analysis (descriptive analysis) and a qualitative analysis (discourse analysis, following the principles of grounded theory).

The Twitter Archiving Google Spreadsheet 6.1.7 (TAGS) application was used to gather data. The SPSS Statistics package, version 24, was used for statistical analysis, and Atlas Ti HM software, for discourse analysis.

### 4.3 Methodology

The process began with the configuration of the Twitter Archiving Google Spreadsheet 6.1.7 (TAGS) for the download and configuration of all the tweets which had the tag #EsteVirusLoParamosUnidos. At the end of the data collection period the Excel sheets, with all the data bases, were downloaded. Four Excel sheets were obtained with the following data:

1) ‘Read me Settings’: Here start and end dates of the download process are identified.
2) ‘Summary’: This section provides information about the top tweeters and the number of tweets sent by these users. In addition, the following was extracted from this sheet: number of links, number of retweets, number of unique messages sent and other complementary data such as the number of responses with @s.
3) ‘Dashboard’: This consists of graphs showing the evolution of the conversations in real time.
4) ‘Archive’: In this section all tweets are downloaded. Each tweet has: the user ID, the text of the message, the creation and sending date, the sending source (Hootsuite, Twitter web App, Twitter web App for Android or iPhone, etc.), URL of the Twitter account, the number of followers and users it follows, the location of the account, etc.

**Figure 1:**
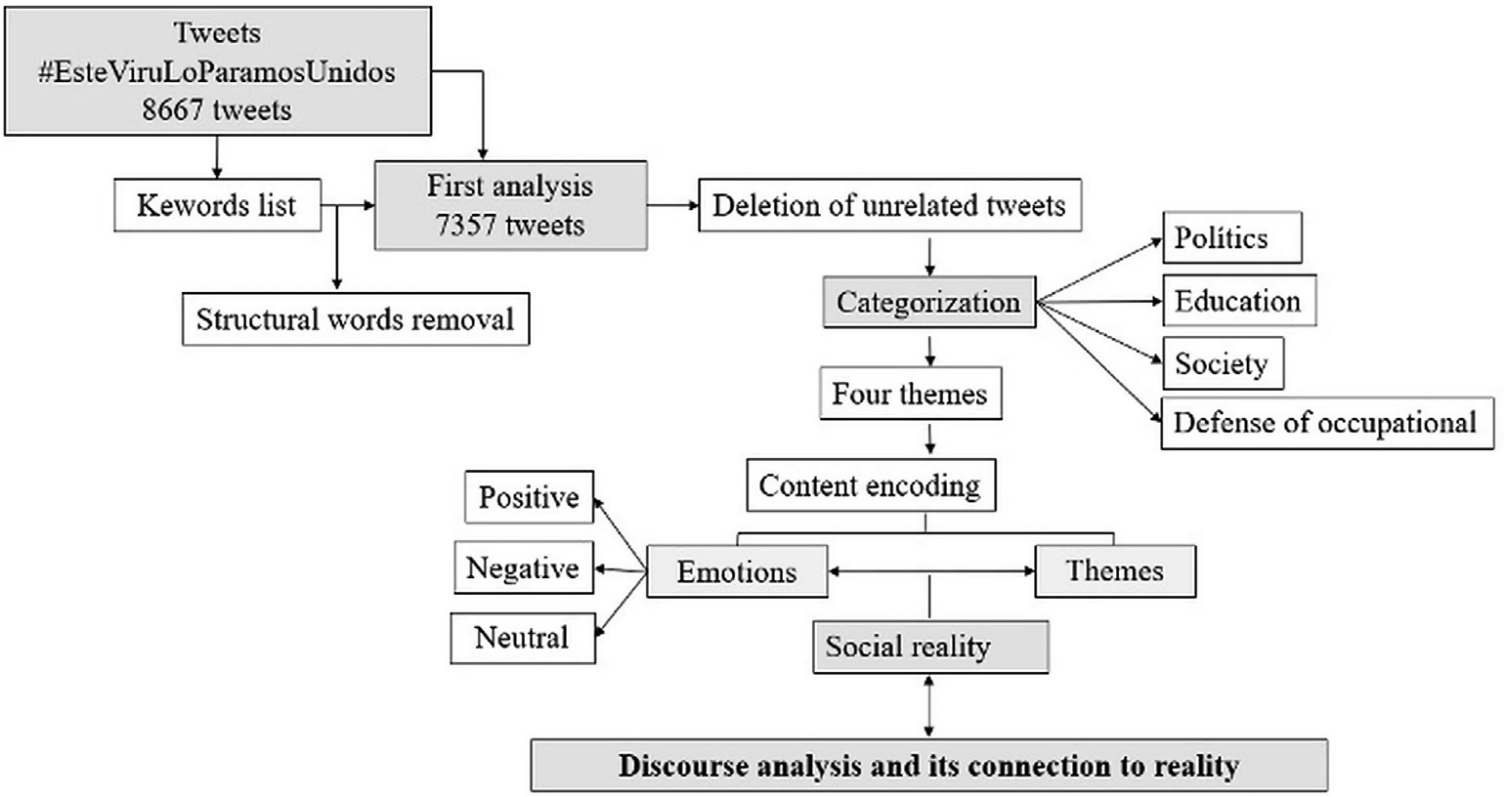
Discourse analysis process.

For the quantitative analysis, the ‘Summary’ sheet has been taken into account while, for the qualitative analysis, the data from the ‘Archive’ sheet was used. The descriptive analysis was used to extract frequencies of participation and the method of communication used in the discussion (tweets sent, retweets, linking). After the thematic coding was completed, the main themes involved in the public deliberation were subjected to frequency analysis and percentage analysis. No automatic data procedure was used. A manual data processing was carried out for the discourse analysis. Figure 1 shows the steps which were followed for the analysis of the emotions expressed on Twitter.

The method chosen for this research was discourse analysis. Discourse analysis is highly relevant in this kind of research where a large amount of information is processed. This information included participants with diverse profiles and when posts, written in colloquial language, prevailed. Discourse analysis [66] was used to study the general feelings expressed by citizens during public deliberation, the prevailing emotion associated with the identified topics, and the connection between discourse and the social reality experienced at the time. The analysis was carried out in three stages [67, 68]: definition of the content universe and sample collection, selection of the unit of analysis and, lastly, establishment of categories and codes.

The procedure for the categorization and coding was done using Excel. Each tweet was read and coded using Excel according to the theme and emotion expressed. In those tweets accompanied by a URL, the tweet was accessed in order to establish the complementary content. The categorization and coding were undertaken manually.

For the codification of the text, the indications of Gibbs [69] and Flick [67] was followed. These authors believe that coding is ‘indexing or categorizing the text in order to establish a framework of its thematic ideas’. The treatment of data, through coding, involves identifying and recording one or more phrases from the text which describe the same idea. Once these ideas are identified they are linked to a code or name. Dey [70] claims that this naming is not arbitrary since it is developed by means of a careful and thoughtful process of categorizing content.

First, prior to coding, a previous reading of the tweets was carried out. A total of 1,310 tweets were discarded as they were not directly related to the campaign and failed to offer relevant information. To carry out this first immersion, Atlas Ti software was used to facilitate the identification of the most frequently-used words and allow for a first orientation on the topics covered in the selected tweets. Those words with a structural function in the Spanish language (que, de, el, la etc.) were discarded. However, those words, which offered relevant information, and could consolidate the type of communication used by society, were retained.

From this list a template of potential codes was built, following [71] who recommends, for its construction, ordering the potential codes hierarchically. To prepare this list some specific criteria were taken into account, such as grouping of similar terms, variations with the same meaning and the selection of terms based on their frequency of use. Here, words with a frequency of greater than 50 repetitions have been selected.

By means of a second, more careful, reading, an analytical and axial coding procedure was followed, which facilitated the organization of the data into categories and subcategories [72]. New ideas were grouped in global codes that included more specific codes [69] began to be detected.

Finally, two main categories were established: a) thematic and b) expressed emotion (Figure 1). Two levels of coding were established for each category (general and specific), as illustrated in Table 1. From the category ‘Thematic’, four general codes were extracted: ‘Politics’, ‘Messages to society’, ‘Defence of occupational groups’ and ‘Education’. Within the ‘Politics’ code there are messages both for, and against, the current Spanish government. They include messages of protest and complaints about the lockdown measures implemented by the government. In the ‘Messages to society’ code, users were seen to strengthen ties of union, expressing encouragement and gratitude towards citizens, seeking social cohesion and providing relevant information aimed at defeating the common enemy: the virus. In the third general code, ‘Defence of occupational groups’, citizens express their gratitude to the state security forces as well as to the scientists and health workers who played a fundamental role in this health crisis. Finally, ‘Education’ includes, in the majority of its messages, information on regulations implemented by schools. This relates to measures that students had to take in order to return to their classrooms during this exceptional period.

**Table 1:** Coding of the categories ‘Themes’ and ‘Emotions’.

Category	General codes	Specific codes
Themes	Politics (Po)	Deniers (Ne)
		Criticism and social anger (Cr)
		Political parties (Pol)
	Defence of occupational groups (De)	Health and science (H)PH Law enforcement (D)
	Messages to society (NS)	Official data (OD) Prevention and health measures (PH) Social cohesion (SC)
	Education (E)	Education (E)
Emotions	Positive (P)	Unity (U)
		Support (Ap)
		Optimism (Op)
	Negative (Ne)	Discontent (Des)
		Criticism (C)
	Neutral (N)	Objective information (IO)

With the aim of examining these general codes, the most frequent-used words extracted from the 7,357 tweets in the sample were reconsidered, focusing on the most repeated concepts in each of the four general codes (‘Politics’, ‘Messages to society’, ‘Defence of occupational groups’ and ‘Education’).

This step, together with a new detailed reading of the messages, served as the basis for defining a new level of specific encoding. Nine new codes were specified which convey specific information about the debate generated by the Twitter messages (‘Negationists’, ‘Criticism and social anger’, ‘Political parties’, ‘Health and science’, ‘Law enforcement’, ‘Official data’, ‘Prevention and health measures’, ‘Social cohesion’ and ‘Education’).

In the second category, ‘Expressed emotion’, two specifics levels were considered in order to analyse the feelings exposed in the hashtag. Two aspects were assigned to each tweet: character (negative, positive, or neutral), and feelings, where we found some positive sentiments (unity, support and optimism) and some negatives (discontent or criticism). Those messages, categorised as neutral, were perceived to convey ‘objective information’. Once these values were assigned to each of the 7,357 messages, a quantitative and qualitative analysis of the frequencies of each of these emotional categories was carried out. This allowed us to explore, in depth, the feeling of citizens regarding the #EsteVirusLoParamosUnidos campaign. On completion of the coding, the frequencies and percentage of each code were established.

Once the entire exposed process had been carried out, five concept maps, relating to the topics which appeared on Twitter, were developed. The first of these maps includes information on the main codes, while the other four refer to each of these codes. The concept maps were made using Atlas Ti which helped establish the connection between the concepts used by Twitter users in this campaign.

These maps have three levels of precision. Firstly, they illustrate the seven general codes extracted from the public debate generated on Twitter. Secondly, they show the corresponding specific codes and, thirdly, they give information on some of the most frequently-used words. The frequency and density (number of codes with which it is related) of the most frequently-used words are indicated on the maps, following the formula: concept + number of repetitions in the text: number of codes with which it is related (Ex.: Police 708:4). Based on this data, and after close examination, a description of the public debate, generated around each of these codes, was developed. This was in addition to the connection between citizens’ discourse and the social reality experienced at the time. Some examples, taken from the most representative tweets, are included specifying the order number according to the publication data.

## 5 Results

### 5.1 Communication system: retweeting, direct messaging and quoting

Of the 7,357 tweets making up the sample, 72.32% were found to be RTs, while only 19.74% were direct tweets. Messages with direct quotations using @ (messages in which one tweeter uses the ‘@user’ structure to quote another tweeter) made up a smaller proportion (7.91%). This indicates a low level of interaction and/or response among participants within the #EsteVirusLoParamosUnidos campaign. The data show that 68.62% of the messages included links.

### 5.2 Topics highlighted in the public deliberation

As previously stated, four main codes were extracted from the 7,357 tweets: ‘Politics’, ‘Messages to society’, ‘Defence of occupational groups’ and ‘Education’ (Figure 1).

‘Messages to Society’ was the most discussed topic (65.07% of the messages). ‘Defence of Occupational Groups’ was in second place (42.47%) and the final positions related to ‘Politics’ (34.18%) and ‘Education’ (3.97%). Each of these main codes was coded (as shown in Table 2), and generated nine specific codes:

**Table 2:** Theme analysis.

General	Specific	%	Total
Politics	Deniers	1,29%	
	Criticism and social anger	7,34%	12,6%
	Political parties	91,37%	
Defence of occupational groups	Health and science	12,29%	
	Law enforcement	72,6%	33,29
Messages to society	Official data	31,8%	
	Prevention and health measures	61,3	51%
	Social cohesion	6,9%	
Education		–	3,11%
Total			7357

Within the code ‘Politics’, there are the following specific codes:

- Deniers: Messages from users who disbelieved in the existence of the virus, holding that the Covid-19 pandemic was an invention of governments who use fear as a means of crowd control.
- Criticism and social anger: In these messages, users express their anger and dissatisfaction with the situation created by the pandemic. They blame politicians for having failed to legislate with citizens in mind. In addition they express their anger from a personal perspective, explaining how the virus had affected their businesses or their families.
- Political parties: These messages refer to certain political parties and highlight two different perspectives: on the one hand, messages from users who supported the government, led by Pedro Sánchez. On the other hand, messages from users who were against the government - in other words, supporters of the opposition.

‘Messages to society’ has three specific codes:

- Official data: These messages contain data, promoted by official sources, in which information related to the spread of the pandemic, was transmitted (number of deaths, legal measures adopted…)
- Prevention and health measures: Users who posted this type of message encouraged citizens to respect security and health lockdown measures. Most of these messages are simple reminders of what could be done, individually, to overcome this collective health crisis.
- Social cohesion: This category includes those messages which promote social cohesion. They express encouragement and gratitude to society as well as positive thoughts about the advance of the pandemic.

Thirdly, within ‘Defence of occupational groups’ we find two specific codes:

- Health and science: Users who showed their support, and trust, in health professionals and doctors who have fought to care for the most vulnerable people and scientists who have searched for the best cure to stop the spread of the pandemic.
- Law enforcement: Users participating in this campaign also expressed their gratitude to the state security forces. Thanks to them, they felt safe and proud of their country.

Finally, the ‘Education’ code has a single code at this specific level:

- Education: Messages referring to the educational field. These were mostly posted with the aim of transmitting information on the preventative measures introduced in Spanish schools.

### 5.3 Emotions expressed in public deliberation

Qualitative and quantitative analysis of the feelings expressed in public deliberation as part of the #EsteVirusLoParamosUnido debate, shows that emotions followed a continuum which created two classification levels. The positive, negative or neutral character of every tweet regarding the social reality which was being experienced was taken into consideration, in order to analyse, in depth, the sentiment expressed in each testimony.

First, positive messages were found to be steeped in feelings of unity, optimism and support. Conversely, negative messages appeared to express discontent or criticism. Lastly, there were messages with a neutral focus which were limited to the transmission of objective information on the spread of the pandemic around the world. 31,5% of the messages analysed show a negative disposition and express critical feelings (7,9%) or feelings of discontent (23,5%). Despite the challenging situations experienced since the beginning of the pandemic, 27,9% of the messages were positive, expressing feelings of unity (18%), support (8,4%) and optimism (1,6%). A larger proportion (40,6%) were neutral with the purpose of transmitting objective information about the Covid-19 crisis.

The frequencies resulting from the quantitative analysis of feelings are shown in Table 3. This kind of analysis offers a more detailed understanding of the emotions expressed on Twitter, and connects with the citizens’ analysis discourse presented below.

**Table 3:** Feelings expressed.

Character	Total	Feelings	Total
Positive	2050	Unity	1,324
		Support	620
		Optimism	112
Negative	2319	Discontent	1,731
		Criticism	584
Neutral	2988	Objective information	2,986

‘**Unity**’: This includes messages which encourage people to prevent the spread of the virus, indicating that it was everyone's responsibility to be aware of the prevention measures with a view to minimizing community contagion. In addition, there are messages revealing the actions that some organisations (private companies, security forces, etc.) carried out in order to contribute to the public good (‘Take care of yourself by taking care of others’ [T2526]; ‘Together we will get it’ [T3372]).

‘Support’: This is a feeling shown by citizens especially towards the most vulnerable groups in society, in particular older citizens or the disabled. They offer support in times of pandemic. Numerous examples of supportive messages were also gathered for the state security forces [T3968], scientists [T3677] and health workers [T3604].

‘**Optimism**’: Includes messages of encouragement and hope, highlighting the small achievements and improvements in public health. There are messages celebrating the possibility of returning to some face-to-face meetings which were abstained from during lockdown (‘today, after such a long time without face-to-face lessons, we are starting a new course’ [T2014]).

‘**Discontent**’: These are messages of anger or frustration at the restrictions derived from the health situation caused by Covid-19, in addition to messages of protest unrelated to health issues, such as the trade union demands of the JUSAPOL platform, which, to a great extent, used the hashtag to promote their campaign for ‘equal pay’ [T819].

‘**Criticism**’: Include messages which appear that express discomfort towards a number of different scenarios caused by the pandemic. Their anger is focused on a particular entity, such as a political party or a specific company (‘This government wants to destroy the economy of Spain’ [T7253]).

In studying the emotions generated in each category, we have observed that messages falling within the ‘Politics’ category express criticism and discontent in response to the situations users experienced due to Covid-19, and in response to how politicians have dealt with pandemic-related situations (‘The calibre of our politicians here in #Spain is ‘unreal!’ Cringing so hard I've had to stop. It's harmful to your intelligence...’ [T2332]). Arguments were waged between tweeters who took up political or ideological positions, sometimes expressing hostile or aggressive views (‘The numbers show how wrong @IdiazAyuso is. As irresponsible as she is stubborn’ [T6137]; ‘It's always the same with Communists; first they lie, and then they say it's our fault……’ [T4461]).

Communication style, in the category ‘Messages to Society’, highlights a reconciliatory tone fostering social cohesion. They are messages which encourage a feeling of cohesion throughout Spanish society, sought to bring people together in the fight against the common enemy of the pandemic (‘Viruses don't discriminate. They don't see origins or ethnic groups. We people shouldn't, either…’ [T4503]; ‘The battle against #Covid_19 will be long, but we'll win it with empathy and unity…’ [T3860]).

The category, ‘Occupational Groups’ contains messages of unity defending the public sectors and asserting workers' rights. They are critical but polite, proclaiming support in their promotional posters, which call for help to withstand the Covid-19 crisis. ‘Health’ and ‘Science’ are the fundamental pillars (’@IreneMontero - Why don't you fight for members of the Civil Guard or the Police in order to earn the same as members of the Mossos or Ertzainas? Liars…’ [T383]; ‘Pharmacists release an urgent manifesto defending their role in the fight against Covid-19…’ [T1906]; ‘Jobs need tourism…’ [T351]).

Lastly, the ‘Education’ category focuses on a functional discussion with an objective, informative style of communication to talk about measures and data concerning the return to face-to-face teaching and new ways of socially distancing in educational establishments. This category includes messages supporting the education sector as it braced itself for the reopening of schools.

(‘Are we well informed about the precautions we have to take in order to avoid classroom infection? Here's our Basic Prevention Guide for Schools…’ [T933]).

### 5.4 Analysis of the connection between discourse and the social reality

The next step was to explore how the discourse generated around the Ministry of Health, Consumer Affairs and Social Well-being's #EsteVirusLoParamosUnidos hashtag. This was linked to the social reality experienced by Spanish citizens in September and October 2020. Within this context we established four main categories: ‘Politics,’ ‘Messages to Society,’ ‘Defence of Occupational Groups’ and ‘Education,’ which are made up of specific significant contents and messages. Their links with social reality are analysed, in depth, below.

The first analysis was developed in order to study the discourse generated around the hashtag #EsteVirusLoParamosUnidos. It involved the categorization of the 7,357 selected tweets in the four categories previously mentioned (‘Politics’ ‘Messages to Society’, ‘Defence of Occupational Groups’ and ‘Education’), focusing on the relevant content of the codes analysed. By analysing the topics, which were addressed the most, within the general codes, a second level of precision was obtained (Table 1).

In addition to the new codes (described above), a useful technique in this process was an analysis of the most frequently-used words extracted from the 7,357 tweets. From this analysis of frequently-used words, we see that, in ‘Politics’, 9 words stand out as they refer to the main political parties in Spain (Figure 3). In the next category, ‘Messages to Society’, 10 concepts have been selected in order of frequency (Figure 4). ‘Defence of Occupational Groups’ has fourteen relevant words (Figure 5). Finally, the category ‘Education’ has a fewer number of impact terms, since there are four terms with a frequency greater than fifty repetitions. The analysis of the most frequently-used words guided us to a third level of precision, which explored the main focus of interest of the participating users.

**Figure 2:**
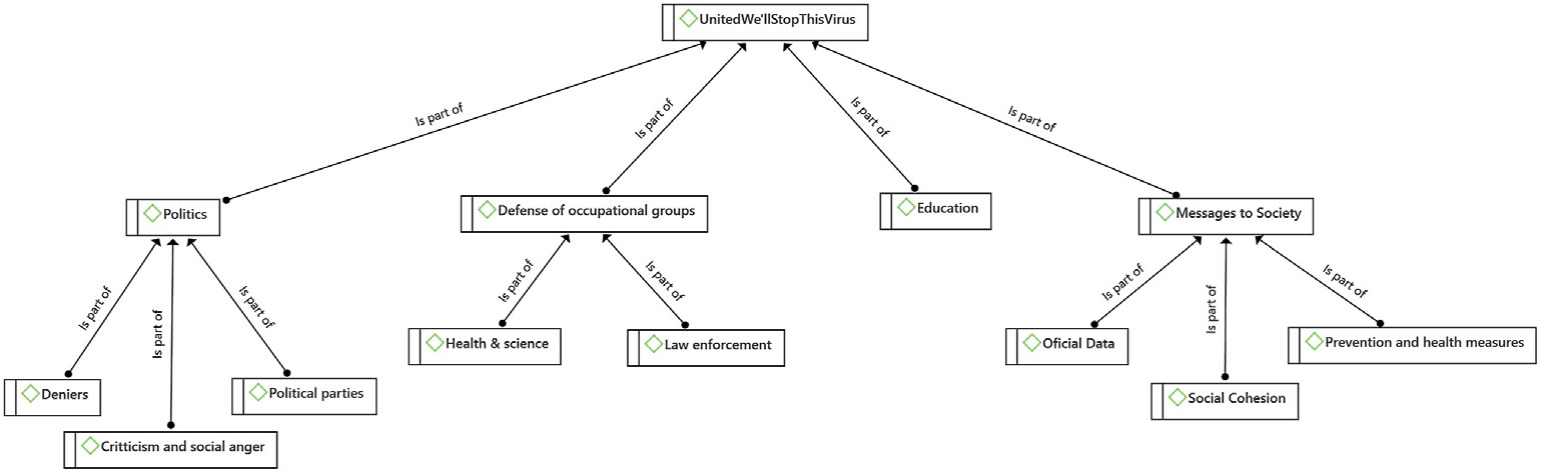
Overall concept map of #EsteVirusLoParamosUnidos showing three levels.

**Figure 3:**
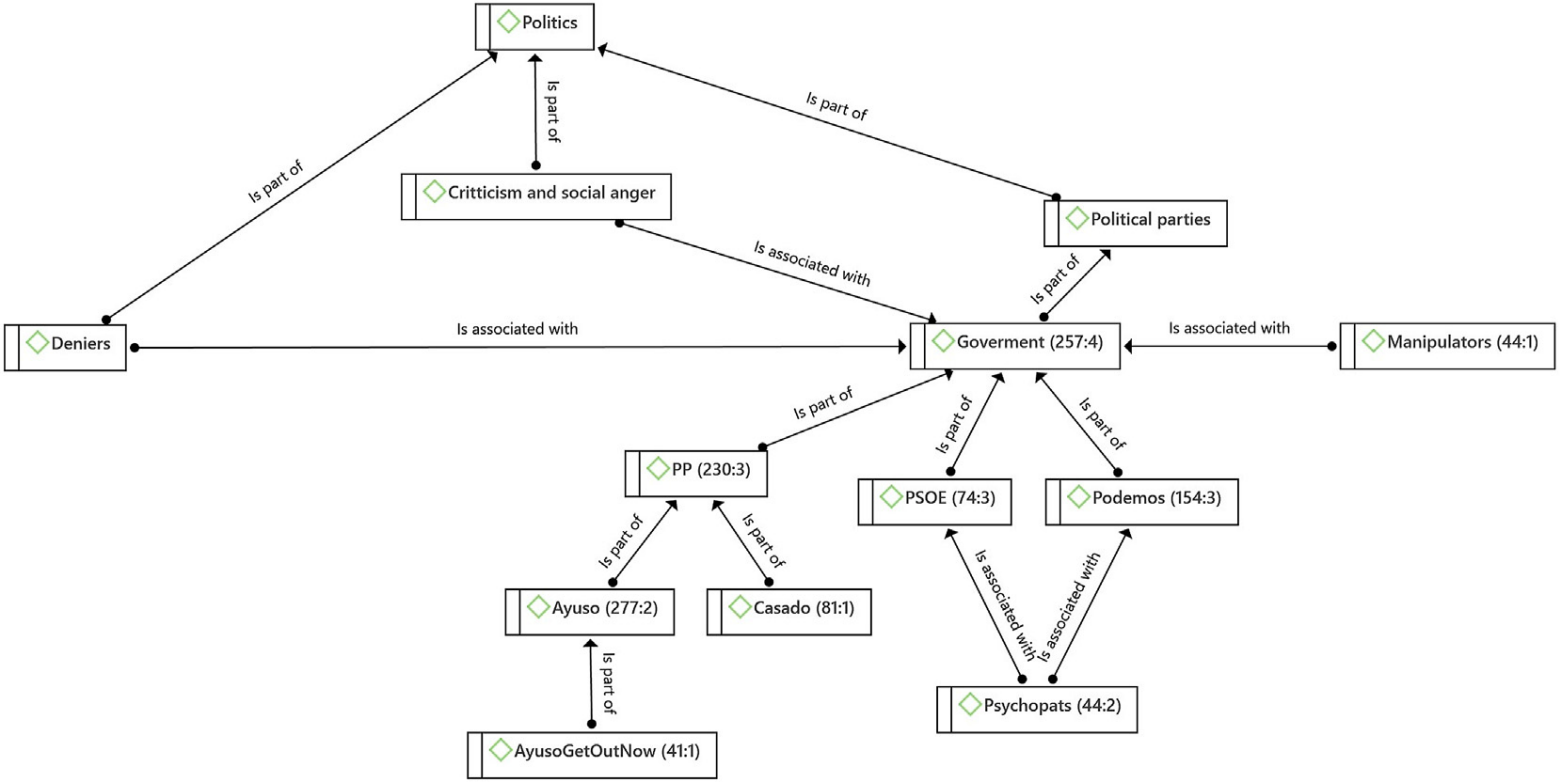
‘Politics’ category.

**Figure 4:**
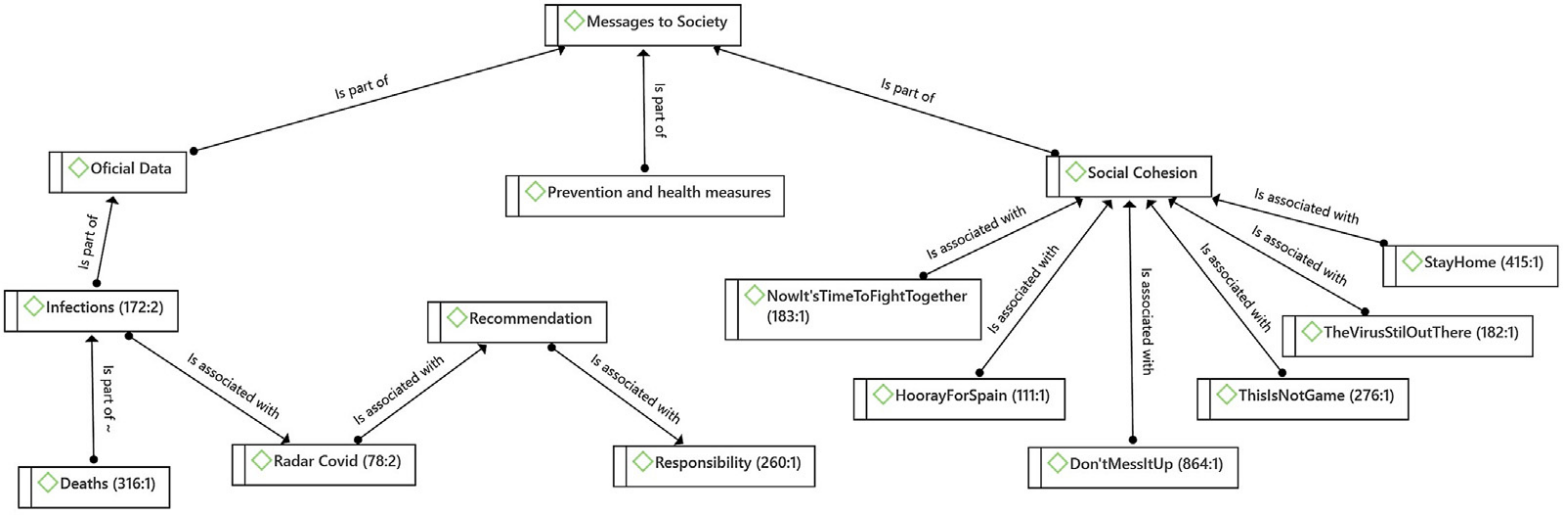
‘Messages to Society’ category.

**Figure 5:**
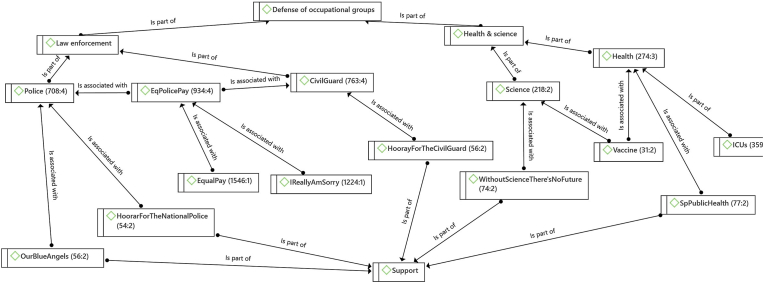
‘Defence of Occupational Groups’ category.

**Figure 6:**
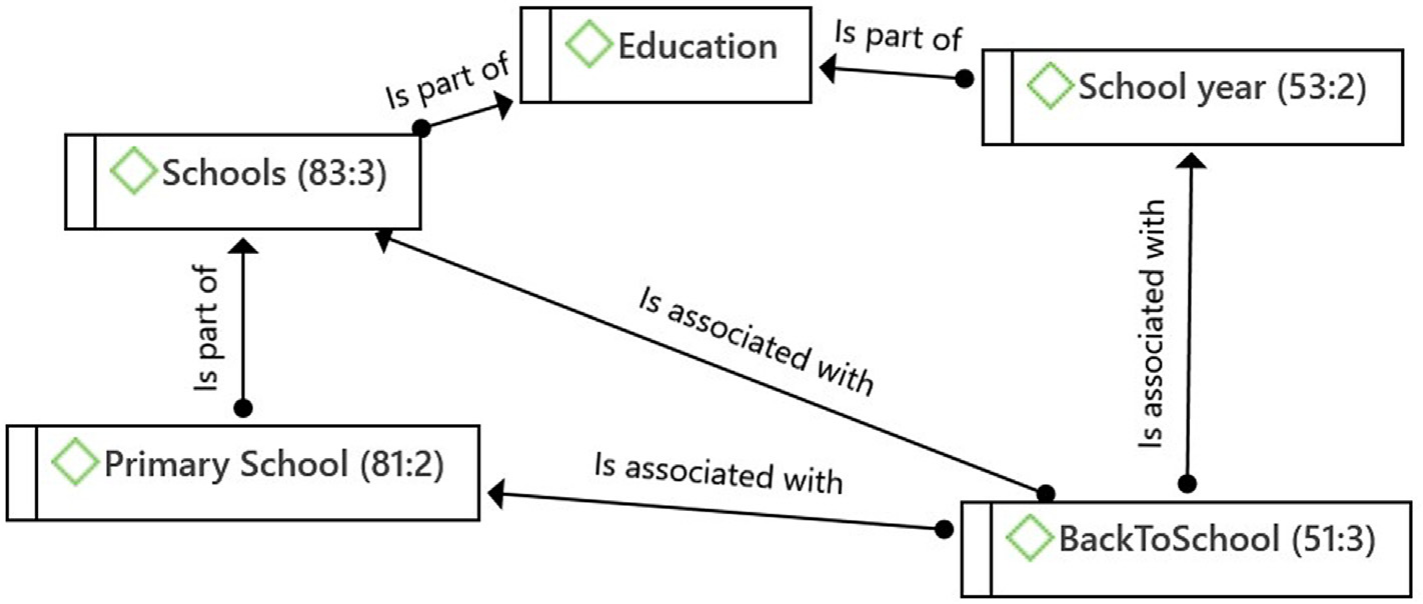
‘Education’ category.

Five concept maps were developed. First, there is a general map, which addresses all the main themes, or codes, extracted from the 7,257 messages (Figure 2). Second, there are four specific maps, one for each of these main codes (Figure 3; Figure 4; Figure 5; Figure 6), referring to the categories of analysis. Here both the frequency and the impact of each concept are addressed. This process was undertaken with the support of Atlas Ti, exposing the existing links between the different themes involved.

The ‘Politics’ category contains most of the messages addressed to the leading governing, and opposition, political parties, PP, Podemos and PSOE. Arguments waged back and forth from the various party-based political positions, and party leaders were referred to directly. Among the messages of support for the government, led by Pedro Sánchez and the PSOE-Podemos coalition, some are brief and concise. They include expressions of satisfaction with the current government and its leaders. Nevertheless, most messages of support for, and trust in, the coalition government include ironic or hostile messages aimed at opposition parties. There are also messages about the privatization of healthcare by the Partido Popular in previous legislatures and direct references to the President of the Community of Madrid, Isabel Díaz Ayuso. This triggered the greatest backlash (‘Defenceless people of Madrid are suffering under the Bolivarian red/Podemos dictatorship in a cruel state of alarm. It's a mess. #AyusoResign…’ [T2421]; ‘Never forget the ravages of the PP in relation to Public Health…’ [T2436]).

Tweet T2436 shared a 1 min and 51 s video alternating between Spanish television news reports on the Partido Popular's privatization of healthcare initiatives with sound bites from the Partido Popular's general secretary, Pablo Casado, criticizing the Spanish government's handling of the Covid-19 health crisis. The ‘Politics’ category also contains messages from tweeters who supported the opposite stance. They rail against the current government with hostile messages calling members of the government ‘manipulators,’ ‘Mafia goons’, even ‘psychopaths.’ Some refer to a newspaper article, written by Pilar Díez [73], and published by Libertad Digital, which reviews an assortment of the main headlines and opinions which appeared in Spain's most influential newspapers (El Mundo, El País, ABC and La Razón) about the handling of the pandemic during the early stages of the lockdown.

‘Messages to Society’ features prevention as one of the core ideas. Tweets encouraged responsible behaviour and shared advice on combating Covid-19, primarily in terms of implementing safety measures (‘Masks are mandatory Stay a safe physical distance from others. Wash your hands frequently. Limit the number and frequency of your social contacts [T1796]). There is information issued by official sources (national government authorities, local government authorities, unions, etc.) reporting on the spread of the pandemic in different regions, cities and towns (@CiudadDepGc reminded citizens that everyone had a part to play by complying with preventative measures [T2843]’). A large number of messages of support, encouragement and group feeling sought to foster hope and citizen unity in the face of the health crisis through social cohesion. ‘We have a lifetime to remember that, in hard times, when standing firmly together, Spain was a great country.’ [T3524]; ‘the strains of ‘Resistiré’^1^ are the soundtrack of the #CollectiveApplause^2^’ [T271]; Thank you for taking care of us.’ [T990]).

The data also reveal initiatives and social acts undertaken by private institutions in order to thank society for making an effort during lockdown. One example is the appreciative statement which was issued at the headquarters of the Vocento communications group on 24^th^ September, 2020. This is when the group's CEO, Ignacio Ybarra, said, ‘The immense wave of solidarity that's flooded our country is surely the best antidote for beating the virus' [74].

The ‘Defence of Occupational Groups’ category, in the third place, contains messages intended to highlight the importance of certain occupational groups in society. These are messages of support from different organizations and private individuals who praised the work of professional groups and called for aid to help pull through the Covid-19 crisis. The messages place special emphasis on science and health, viewing both as fundamental pillars in the fight against the pandemic. Here there are three main foci: national law enforcement, health, and science.

The tweets in ‘National Law Enforcement’ category consist of citizens' messages encouraging, and thanking, the various national law enforcement organisations, and messages posted by members of these occupational groups (‘local and municipal police work to give you safety 24 h a day, 365 days a year. #WeWorkToProtectYou…’ [T5912]; ‘#OurBlueAngels …’ [6179]; #OurGreenAngels [T6181]). One of the high-impact thematic hubs relates to JUSAPOL, an association advocating equal pay for the Spanish Police. Although the themes of these tweets are not directly related to the Spanish government's campaign, their tweeters joined the campaign in order to take advantage of the visibility afforded by participating in the hashtag. Two main thematic foci refer to law enforcement collectives. The first is a demand for equal pay for all national law enforcement agencies, in line with the salaries of the Mossos d’Esquadra. The second consists of messages expressing indignation and protest against the prime minister's expressions of condolence following the death of an ETA prisoner. This upset citizens at the time and associations, like JUSAPOL, called for demonstrations and protests in response on 16^th^ September, 2020, creating the #YoSiLoLamento (#IReallyAmSorry) hashtag. This can be seen from thousands of online messages (‘They're our government's friends. What did you expect? #IReallyAmSorry…’ [155]; ‘ETA will cease to exist when the people the TERRORISTS killed come back to life! #IReallyAmSorry…’ [T6194]).

In the ‘Health and Science’ category we find messages supporting public health initiatives, defending the belief that the quality of the Spanish health system is due to the professionalism of its members (‘The secret: good professionals, good attitude …’ [T1306]; ‘Thanks for taking care of us…’ [T2952]). Reference is made to the hard work of health workers and citizens are called on to respect preventive measures so as to help reduce congestion in hospitals under the catchphrase ‘Cuida a los que te cuidan’ (Take care of those who take care of you). In September, 2020, the National Council of Spanish Medical Associations issued a warning about the health system's critical situation, which it described as ‘borderline’ and ‘overwhelmed’. The council also tweeted its concern over the ‘physical and emotional exhaustion’ experienced by health workers [75]. Carlos Artundo, Director-General of Health for the region of Navarre, voiced his concern over the health of health workers. He said that, in late September 2020, they began to experience ‘chronic tiredness’ [76] (‘Either we all try to do better, or this is going to be a slow death. And health workers are already exhausted…’ [T5530]; ‘There are a lot of us behind each #PCR test...professionals who have switched jobs to keep this #Pandemic in check, in coordination as a #Team. So #TakeCareOfThoseWhoTakeCareOfYou…’ [T5884]).

The ‘Heath and Science’ category also highlights the important work of science and professional scientists. This is interesting because, at that time, high hopes for products, like the Johnson & Johnson vaccine, had just been dashed. On 13^th^ October, 2020, testing of this vaccine was stopped due to an ‘inexplicable illness’ detected in one of the test volunteers [77]. Nevertheless, citizens continued to express their optimism and confidence in the people who were tirelessly fighting and struggling to find an antidote to stop this grave global health crisis. They posted messages like ‘SinCienciaNoHayFuturo’ (WithoutScienceThere'sNoFuture).

The last category, ‘Education,’ refers to the educational sector. Most of the messages in this category relate to primary education. Some show concern for ‘back-to-school’ safety measures for the 2020–2021 school year and present data on the incidence of the virus in the first few weeks of the school term (’...don't forget your Covid Kit. Wear a mask and carry a spare. Disposable tissues. Hand sanitizer. A bag or belt pouch to carry it all in…’ [T3449]; ‘…a hundred students placed in isolation in just one day…’ [T5672]; ‘…we invest in a range of resources to ensure that families can send their children to school with peace of mind…’ [T4420]).

Furthermore, soon after digital technologies were heralded as the perfect way to keep classes running safely during lockdown, messages on the switch to digital teaching appeared, together messages about the need to adapt to on-line instruction (‘Chambers of commerce offer free webinars on new technologies, #digitalization and #innovation to adapt to the current situation's needs…’ [T4648]).

## 6 Discussion

In order to combat Covid-19, the Spanish government launched the #EsteVirusLoParamosUnidos campaign, which aimed to solve this public health problem by means of citizen cooperation and collaboration. The medium chosen to launch the campaign (Twitter) demonstrated an increase in the value the government placed on the importance of citizen participation in drumming up support for their measures. The impact of Twitter is impossible without citizen cooperation. In line with other research [42], the analysis of emotions shows that the predominant feelings expressed online were positive or neutral. On the one hand, we conclude that citizens’ general views about the campaign #EsteVirusLoParamosUnidos, were positive, mainly encompassing feelings of unity and support in the face of the social reality experienced as a result of the Covid-19 health crisis. However, there were many messages of a marked negative nature. These are seen to convey feelings of discontent and anger at the pernicious consequences which citizens experienced at a professional level, and also messages which criticise the management of the crisis by both national or regional governments.

Even though the quantitative analysis revealed a predominance of negative feelings, most of the negative messages appear different from the nature of the hashtag. This reflects the clear intention of certain groups, and companies (for example the JUSAPOL collective), to take advantage of the success of the platform's campaign, in order to benefit their digital profile. Therefore, polarized feelings on the part of the citizens participating in this campaign were identified. This reflects the reality of Spanish society which has been accentuated during the Covid-19 pandemic [78].

Spanish society's tendency to post positive messages is consistent with recent research carried out during 2020 [79,80,81]. However, an analysis of the debate, based on a longer period of time, would provide us with a more comprehensive mapping of emotions [22] as the tendency to express negative emotions as the Covid-19 situation worsened.

On the other hand, we found a number of neutral messages which lacked emotional nuance. Their purpose was the transmission of objective information (geographical restrictions, information on contagion prevention measures, reopening of companies, etc.) taking advantage of the fact that hashtags are a tool which focus attention and debate on the tagged topic [82].

This communication system has focused on spreading two key messages. From an objective perspective, it gave information on infection rates and offered health and safety advice, etc., in order to alleviate the effects of the pandemic. Secondly, it has served the purpose of supporting certain political ideas and social demands (defence of public sectors, equal pay for law enforcement agencies, etc.). The communication process focused on the use of RTs which indicate a low level of interaction and/or discussion. Basically, the Spanish government has used the Twitter network to spread information more than to interact with citizens [27]. However, our conclusion is that the campaign has achieved its objective: ‘to unite the efforts of the entire country in the fight against Coronavirus and to provide incentive for social distancing measures’ [65]. Citizens answered the call by retweeting. New ways of connecting, and patterns of interaction, were generated between the Spanish government and its citizens via Twitter [3]. However, they were based, primarily, on spreading information about the health safety measures which the government wanted the population to embrace, rather than establishing a true exchange.

The themes of this discussion are of great political, economic, and social importance, and of interest to all of society. They highlight the impact of communication between citizens and the political agenda [5]. Four main thematic hubs have been identified: ‘Politics’, ‘Messages to Society’, ‘Defence of Occupational Groups’ and ‘Education’.

The content of the tweets shows a clear connection between the discourse of the citizens who participated in public discussion and the social reality they experienced at the time of tweeting. In the first group, ‘Politics,’ the discourse focused on two very different feelings marked by the social context of the period under analysis. On one hand, there were posts from tweeters who sided with messages opposing the government criticising its handling of the pandemic and believing that it was manipulating the media. On the other hand, there are messages of support for the national government. However, they take a stand against measures implemented by the governments of certain regions including the privatisation of healthcare centres in Madrid. This type of confrontation shows a clear division in public opinion - a political and ideological fragmentation which has existed in Spain for decades [83]. This is why unity and cohesion campaigns, like #EsteVirusLoParamosUnidos, have emerged with the aim of raising awareness of common enemies within the population, enemies like Covid-19.

The second theme is termed ‘Messages to Society.’ Here there are glimpses of the social unity which has emerged in Spain in response to the health crisis. This has been shown in gestures like the daily 8:00 pm applause for health workers and the multiple messages encouraging people to keep combatting the virus, reminding them of the measures needed to win the fight. Despite the clear ideological division mentioned above, there is proof that, in the face of major crises, Spanish citizens show amazing solidarity, and seek to help the most vulnerable groups in society.

The fourth theme, ‘Defence of Occupational Groups,’ includes messages from citizens showing support for health workers and scientists, proclaiming the quality of public health as one of Spain's most significant values. In addition, tweeters appreciated and applauded the work of national law enforcement bodies. There are several thematic foci related to these groups which stray from the main theme embraced by #EsteVirusLoParamosUnidos. As previously observed, some tweeters used the hashtag as a means of increasing the visibility of their own campaigns (equal pay, anti-government protests, etc.).

Lastly, content in the ‘Education’ category is remarkably sparse, as it comes from within the context of the resumption of face-to-face instruction during a school year marked by Covid-19-related restrictions.

To sum up, the development of networked social/technical measures [84] and socio-political measures, which enable citizen participation, has facilitated the development of new patterns of interaction between governments and digital citizens. This has increased the possibility of influencing government decisions and the public agenda as well as the opportunity of strengthening citizen engagement in order to cope with health emergencies.

## **7** Conclusions

The general objective of this research focuses on analysing the feelings of Spanish citizens within the framework of a state campaign: #EsteVirusLoParamosUnidos which was promoted by the Spanish Ministry of Health. From the 7,357 messages that make up the study sample, polarized opinions within the population were observed regarding the circumstances experienced as a result of the Covid-19 pandemic. However, we have established a certain trend towards positive views highlighting feelings of ‘unity’, ‘support’ and ‘optimism’. Messages of solidarity and social union stand out as do citizens' support for those health workers who have been fighting Covid-19 on the front line. There are also some negative messages in which users express ‘critical attitudes and feelings of discontent’, because of the numerous social changes experienced during the pandemic. In addition we found messages of anger with, and distrust in, the current government. Taking into consideration the communication system used by those involved in this campaign, the posting of retweets (72.32%) stands out in comparison to the low rate of direct messages (9.91%). This shows a low intention of interaction and debate thus limiting the discourse to the repetition of information of ideas through this social network.

Taking into account the main themes of the public debate, we have concluded that the key topics, addressed in the social network under the hashtag #EsteVirusLoParamosUnidos, were: ‘Politics’, ‘Messages to society’, ‘Defence of occupational groups’ and ‘Education’. ‘Messages to society’ was the category most addressed by Twitter users, with posts referring to the dissemination of official data (OD), posts raising awareness of prevention measures (PH), as well as messages conveying a strong component of social cohesion (CS). Through an exhaustive analysis of the discourse, a close relationship was found between the discourse of citizens via this social network and the social reality experienced on the date their messages were sent.

During the months of September and October 2020, there were some key periods in which social reality underwent many changes as a result of the Covid-19 pandemic. These changes included the effects of political conflict, new restrictions which impacted on different sectors of the workplace, the return to the classroom at the beginning of the academic year and ongoing support for health workers. This is in addition to the different situations in which the Spanish citizens’ feelings of unity and optimism, as observed in our analysis, served as the foundations for hope and inspiration in the fight against a common enemy.

## 8 Limitations and future research

This research provides data of special relevance to the study of the feelings and emotions expressed by Spanish citizens during the Covid-19 pandemic within the framework of a public campaign promoted by the Spanish government. However, it has certain limitations. The main limitation was that we focused on just one of the campaigns, aimed at fighting Covid-19, launched by the Spanish government. Focusing on a single campaign has allowed us to carry out a detailed study, but this has meant a loss of the wider global perspective of the debate in relation to the measures adopted, and the different aspects dealt with, in each of the other campaigns. Secondly, despite the relevance of the time-period which was selected, we did not have the opportunity to explore the emotions expressed by Spanish citizens on Twitter during the final months of 2020 and in the first half of 2021. An analysis of the network debate, based on a longer period of time, would provide us with a more complete mapping of citizens’ emotions during the evolution health crisis in Spain. Future research will be undertaken, thus expanding our sample, as it will include other campaigns, and span a longer period of time.

Finally, an algorithm-based analysis of messages through machine learning models [44] would have facilitated the quantification of emotions and the classification of tweets [85, 86].

## Declarations

### Author contribution statement

Sonia Santoveña-Casal: Conceived and designed the experiments; Performed the experiments; Analyzed and interpreted the data; Contributed reagents, materials, analysis tools or data; Wrote the paper.

Javier Gil-Quintana: Conceived and designed the experiments; Performed the experiments.

Laura Ramos: Analyzed and interpreted the data; Contributed reagents, materials, analysis tools or data; Wrote the paper.

### Funding statement

This work was supported by Universidad Nacional de Educación a Distancia (National University of Distance Education).

### Data availability statement

Data associated with this study has been deposited at Google Spreadsheets under the accession number:

Google Spreadsheets TAG – (continuously updated document): https://bit.ly/3z7ErU1
Google spreadheets TAD (Database): https://acortar.link/KmJPA

### Declaration of interests statement

The authors declare no conflict of interest.

### Additional information

No additional information is available for this paper.

## Footnotes

1 ‘Resistiré’ (I Will Survive), Spanish hit song from 1988.

2 #AplausoColectivo: Nationwide applause which took place every evening thanking health workers and keep spirits high during lockdown.
